# Reversing the Outcome of Synapse Elimination at Developing Neuromuscular Junctions In Vivo: Evidence for Synaptic Competition and Its Mechanism

**DOI:** 10.1371/journal.pbio.1001352

**Published:** 2012-06-26

**Authors:** Stephen G. Turney, Jeff W. Lichtman

**Affiliations:** Center for Brain Science and Department of Molecular and Cellular Biology, Harvard University, Cambridge, Massachusetts, United States of America; University of Cambridge, United Kingdom

## Abstract

During mammalian development, neuromuscular junctions and some other postsynaptic cells transition from multiple- to single-innervation as synaptic sites are exchanged between different axons. It is unclear whether one axon invades synaptic sites to drive off other inputs or alternatively axons expand their territory in response to sites vacated by other axons. Here we show that soon-to-be-eliminated axons rapidly reverse fate and grow to occupy vacant sites at a neuromuscular junction after laser removal of a stronger input. This reversal supports the idea that axons take over sites that were previously vacated. Indeed, during normal development we observed withdrawal followed by takeover. The stimulus for axon growth is not postsynaptic cell inactivity because axons grow into unoccupied sites even when target cells are functionally innervated. These results demonstrate competition at the synaptic level and enable us to provide a conceptual framework for understanding this form of synaptic plasticity.

## Abstract 2

Competition between neurons for the same synaptic sites at the developing neuromuscular junction drives synaptic rearrangements.

## Author Summary

Early in development, neurons make multiple synaptic connections with their target cells. Over time, many of these connections disappear, leaving behind a fraction of the original connections. Because this pruning occurs when mammals first leave the uterus, it's thought that this type of remodeling may serve to sculpt the nervous system to match a particular environment. However, what causes synapse elimination is not well understood. In this study, we use in vivo imaging to study the connections between motor neuron axons and their target muscle cells, at the neuromuscular junction (NMJ), during a developmental stage when each NMJ has multiple connections. We find that synapse loss is driven by competition between nerve cells vying to remain in contact with the same target cell. We show that an axon that would have been eliminated can always be spared by removing (with laser microsurgery) another axon converging on the same synaptic site. The remaining axon not only survives but rapidly grows to occupy the synaptic sites vacated by the removed axon. These results provide a framework for understanding synaptic rearrangements in the developing nervous system.

## Introduction

Physiological evidence that axons completely lose connections with some postsynaptic cells as part of naturally occurring development was first observed at the neuromuscular junction in mammals more than 40 years ago [1]. Since then analogous axonal loss has been seen in many parts of the central and peripheral nervous systems [2],[3]. While the underlying mechanism is still unclear anywhere, evidence suggests that in the neuromuscular system local events at or near the synapse regulate the process. Evidence for local regulation includes the following: (1) the axonal inputs that are eliminated from neuromuscular junctions do so by gradually vacating their synaptic contact sites [4] rather than suddenly undergoing degeneration, as occurs when axons are damaged [5]; (2) the axon that ultimately is maintained increases its synaptic contact area by gradually occupying many of the synaptic sites that were previously occupied by other motor axons [4]; (3) the loss and acquisition of synaptic sites is paralleled by a local reduction and strengthening in synaptic efficacy [6]; (4) the loss of axonal branches from one axon that projects to many muscle fibers occurs asynchronously, suggesting that the timing of elimination is not set by a signal from the cell soma but regulated independently at each neuromuscular junction site [7]; (5) local differences between the synaptic activity of axons converging at the same neuromuscular junction have the ability to cause synapses to be eliminated [8],[9]; (6) local changes in target cell signaling can affect synapse maintenance [10]; and (7) once an axon has vacated all of its synaptic territory at a neuromuscular junction, it locally sheds cytoplasm that is internalized by glia associated with the neuromuscular junction entry zone [4],[11]. Collectively, these data argue that the ultimate identity of the one permanent presynaptic input to a muscle fiber is determined by events occurring at the level of individual neuromuscular junctions. Other data suggest that *neuronal* properties (as opposed to synaptic properties) such as an axon's biochemical identity or its firing pattern play a role in determining the outcome of synapse elimination, but even these may operate through local synaptic mechanisms [12].

Several different local mechanisms have been proposed to explain what drives this process forward. One idea is that individual axon branch removal occurs randomly from a motor unit and is related to an intrinsic requirement that neurons scale back their initially exuberant arbors [13]. A second idea is that the fate of axons is predetermined by positional or perhaps other molecular cues that specify which axon is the best match for each muscle fiber [14]–[16]. A third possibility is that axons converging at a neuromuscular junction compete with each other causing all but the ultimate victor to be removed. It is also possible that some combination of these forces is at play. The idea that synapse elimination is primarily the result of a competitive interaction between the innervating axons was originally proposed because in many muscles the loss of inputs results in exactly one axon remaining at each junction [17].

A competitive mechanism is also suggested by the fact that increases in the size and strength of one input are related to the shrinkage and weakening of other axons [4],[6]. But there is no direct evidence supporting such a mechanism at the synaptic level, and while a number of studies have suggested inter-axonal competition as the likely mechanism, to our knowledge none have shown a direct reciprocal causal relationship between the fates of the surviving and eliminated axons during developmental synapse elimination [8],[18]–[21]. Moreover, in some circumstances multiple axons can remain at the same neuromuscular junction [22]–[24] indicating that in some circumstances either competition can be overridden by other factors or that the whole process is not competitive in the first place. Understanding what drives the process forward is important because this mechanism seems to be one of the strategies at play more generally in the developing mammalian nervous system to help shape it to the particular environment in which it finds itself.

Thus we felt that it would be worthwhile to directly test whether or not synapse elimination is driven by interaxonal competition. We reasoned that if competition between two axons vying for the same postsynaptic site was causing the elimination of one of them, then that axon should not ever be removed if its putative competitor was no longer present. We therefore ablated the axon that had the greatest likelihood of being maintained at a neuromuscular junction to see if the weaker input would have a reversal of fate and now be maintained. If this outcome did occur, we were interested to know when the decision for an axon to be eliminated finally becomes irreversible. For example, it was possible that axons compete and set into motion a program of elimination that is irreversible even many days before the axonal loss finally occurs. The cascade that leads to neuronal cell death has such points of no return [25], which imply that the downstream events are irreversible. Might the same be true for the program leading to synapse elimination? If conversely the synapse elimination program were readily reversible, even at late stages, it would argue that axons remain viable in an ongoing effort to maintain access to the target muscle fiber. In this latter case, the synaptic reorganization events might be played out with little lag between the competitive actions and their consequences on axonal growth or retraction. For example, if one input “pushed” another off a synaptic site, axon withdrawal would be temporally coordinated with near simultaneous axonal takeover, allowing for a highly dynamic process where axonal territory might wax and wane on timescales of minutes or hours. Indeed time-lapse imaging shows that an axon's synaptic territory can be increased and decreased in a dynamic manner [4]. Despite the relatively high temporal and spatial resolution images in many previous studies, however, the motive force for growth and retraction and the details of these behaviors for interacting axons remain obscure.

The experiments reported here allowed us to examine how developing axons respond to vacated synaptic sites. We developed a laser-based technique with which we could remove one of two closely spaced axons that innervated the same neuromuscular junction. This technique showed that axons readily grew to occupy vacant sites even when they appeared to be in the process of withdrawing at the time the sites were vacated. In addition we observed that axons were stimulated to grow even in situations when the muscle fiber was still active. This combination of synaptic vacancy and the axonal takeover it induces allows us to explain a range of complex phenomena associated with synapse elimination.

## Results

### Damaging Axonal Branches in vivo with an Ultrafast Pulsed Laser

In order to selectively remove one axonal branch without damaging any neighboring axons in vivo, we used a diode-pumped mode-locked Ti:Sapphire laser oscillator to cause localized phototoxicity in fluorescent protein containing motor axons in living mice. By taking advantage of non-linear aspects of multi-photon excitation, we could damage one axon and leave immediately adjacent axons unscathed. The focused laser spot was positioned over an axon branch using a modified scanning microscope system (see Materials and Methods and Figure S1), and the axon's fluorescence was bleached at one location. One hundred and seventy-three axons were irradiated (71 in adult neuromuscular junctions and 102 in 1-wk-old neonates).

**Figure 1:**
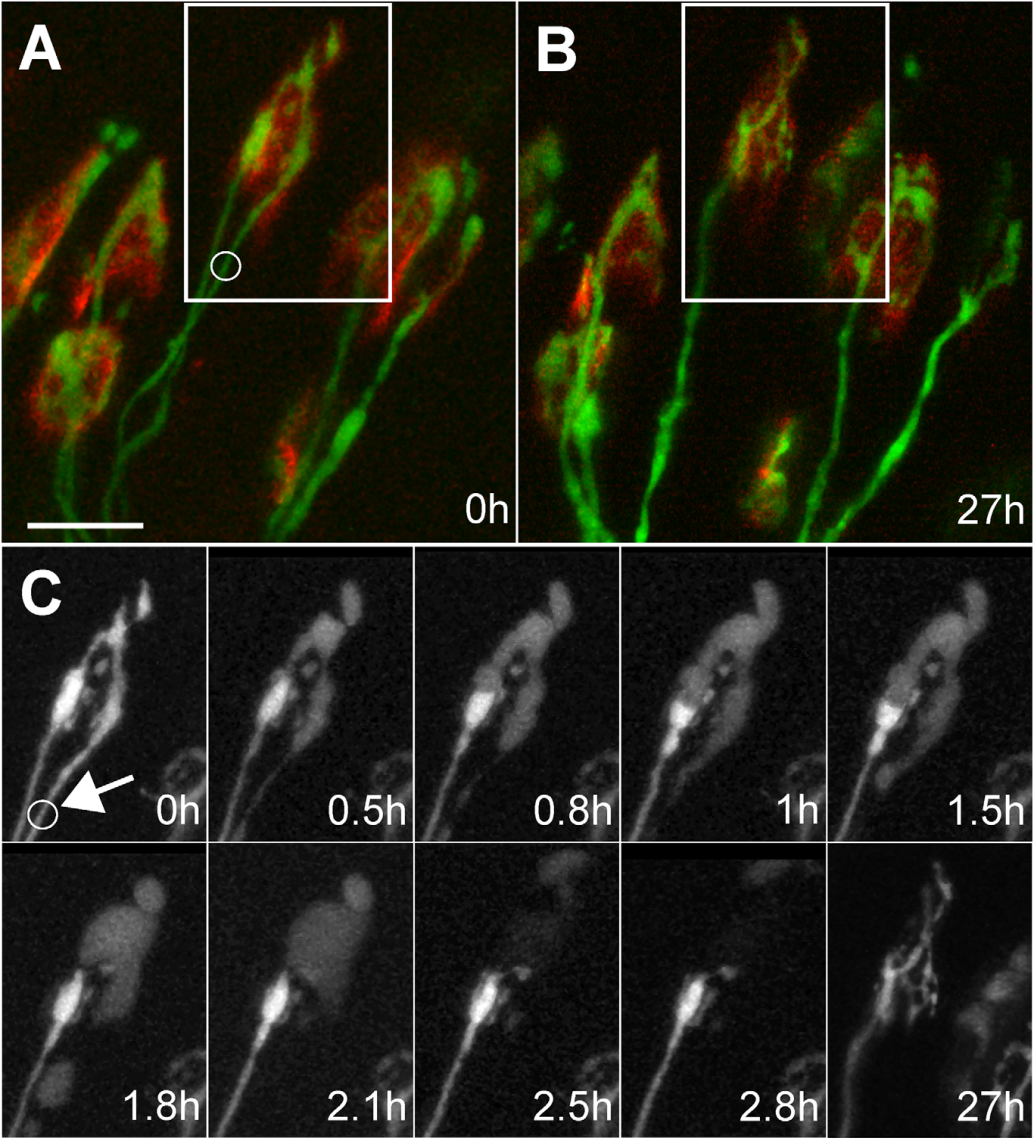
Time course of removal of laser-target input and takeover by another at developing neuromuscular junction. (A) In vivo image of region in a P7 mouse sternomastoid muscle. Boxed area shows a neuromuscular junction innervated by two axons (green, YFP-filled axons; red, α-bungarotoxin-tagged acetylcholine receptors). The axon on the right in the boxed region was irradiated with a mode-locked infrared laser at the site of the circle. (B) The same region of muscle reimaged 27 h later showing that the irradiated axon has completely disappeared. (C) Time-lapse imaging reveals that the remaining axon grows to occupy the sites that were vacated by the damaged axon (arrow points to site of irradiation). Within 0.5 h of irradiation the right axon's terminal branches swell and clearly reveal that the undamaged axon, that remained brightly fluorescent, terminated in a bulb. The swelling of the damaged axon deformed the shape of the intact axon's terminal bulb (panels at 0.8, 1, and 1.5 h). At 1.8 h, the bulb recovered its original shape, presumably because the damaged input had lost its turgor presumably due to membrane leakage. Over the next hour, the fluorescence in the damaged terminals became fainter, its axon fragmented (see 1.8 h), and it largely became invisible. Reimaging the junction 27 h after damage showed that the remaining axon branched to occupy many of the sites previously occupied by the laser irradiated axon. Scale bar, 20 µm.

**Figure 2:**
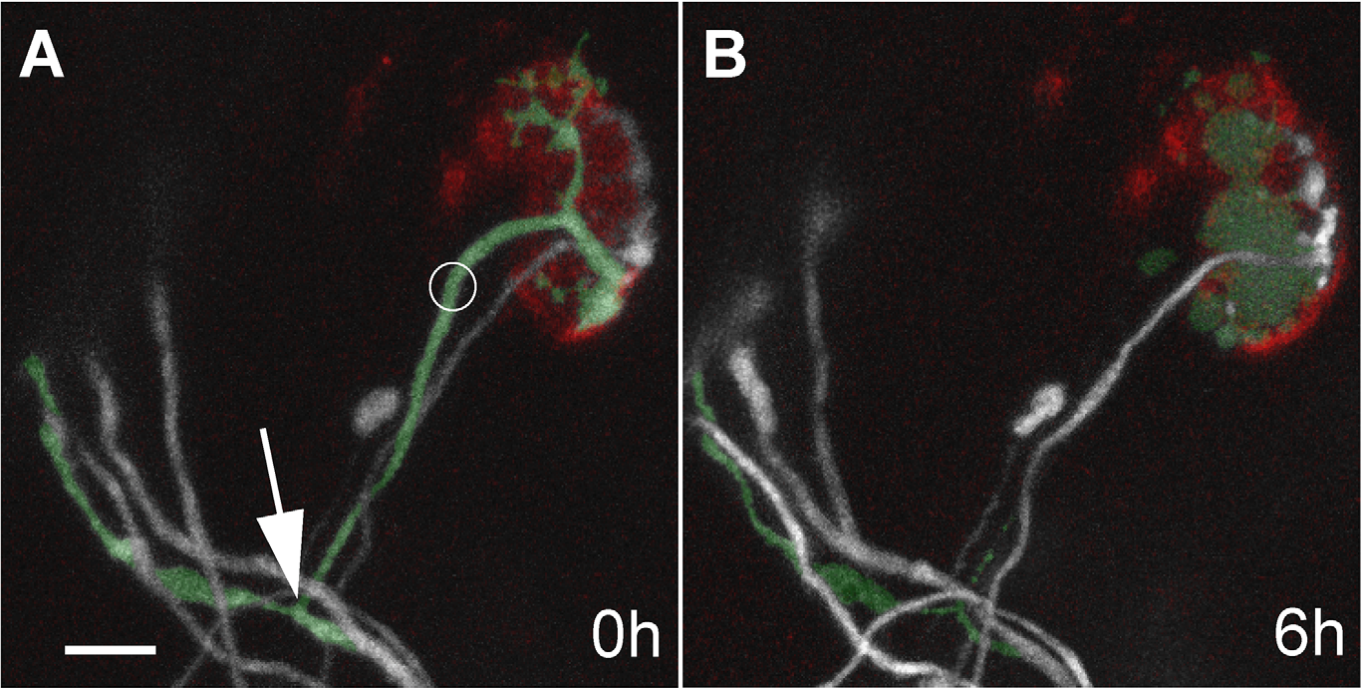
Laser targeted axons die-back to a proximal branch point. (A) To understand the consequences of laser axotomy we irradiated YFP expressing axons at multiply innervated neuromuscular junctions such as the green tinted axon here in a P7 sternomastoid muscle of a living mouse (circle shows site of irradiation). Note that the proximal terminal branch point is visible (arrow). Another axon (grey) with smaller caliber also innervated this junction (and a third recently retracted axon with a bulb ending can also be seen). (B) Six hours after laser irradiation the distal end of the damaged axon swelled, its intensity became fainter, and fragmentation was visible back to the point from which the terminal axon branched off of the parent axon. The relatively small area occupied by the remaining axon is visible at the right edge of the neuromuscular junction site and appears largely unchanged at this time (red, α-bungarotoxin-tagged acetylcholine receptors). Scale bar, 10 µm.

**Figure 3:**
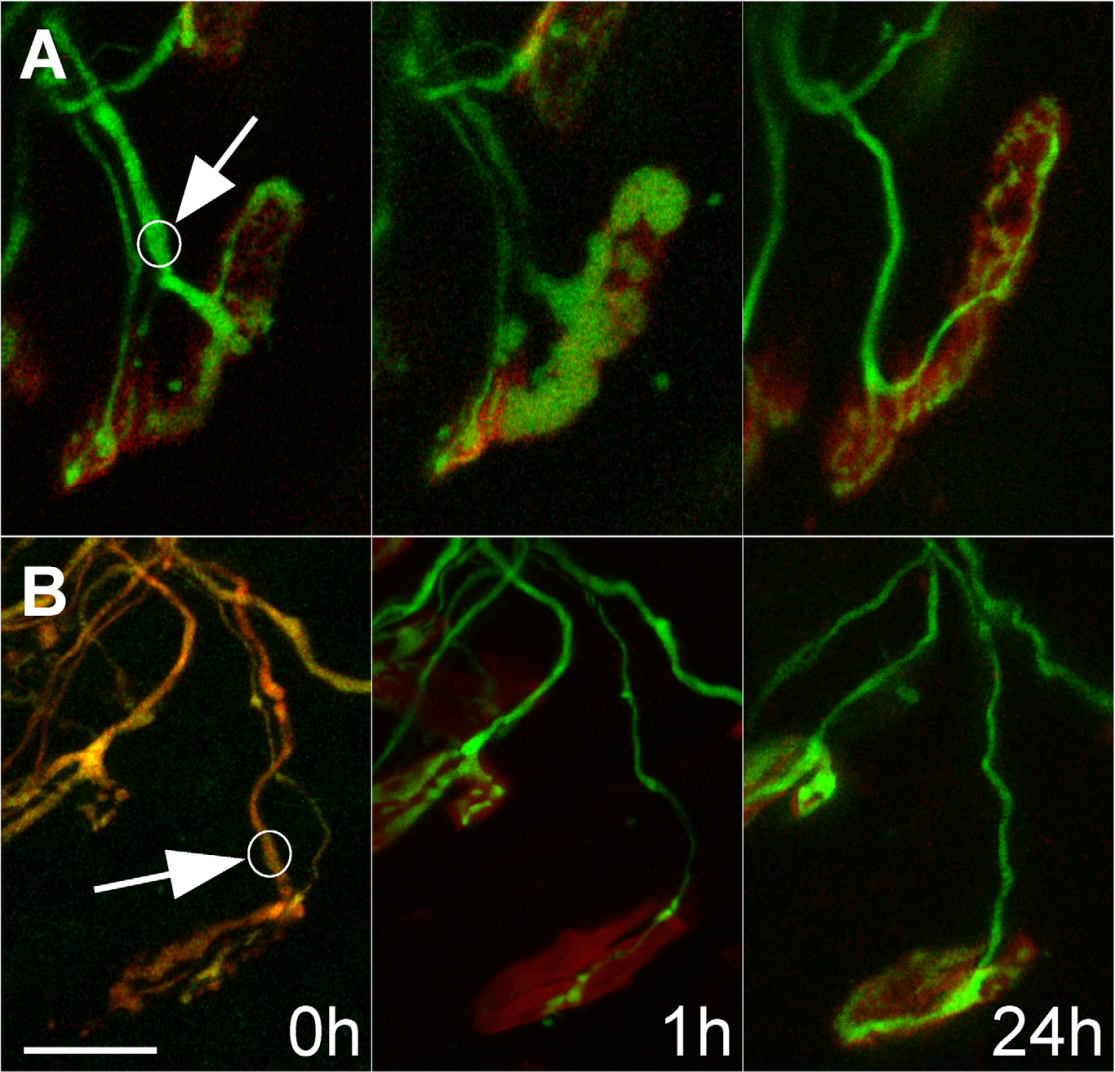
Small axonal inputs take over developing neuromuscular junctions after laser-targeted removal of larger inputs. (A, B) We identified doubly innervated neuromuscular junctions in sternomastoid muscles of P7-P8 mice in vivo, which were innervated by both a small caliber and a large caliber axonal input. Previous studies have shown that in most cases the small caliber input ultimately is eliminated from such junctions. In the cases shown here, laser irradiation of the larger caliber inputs (circles and arrows mark the sites of laser-induced damage) caused (A) their swelling or (B) rapid disappearance within 1 h of laser irradiation. After the damage, the synaptic area occupied by the un-irradiated smaller input could be clearly seen: (a) ∼20% of the total postsynaptic territory (lower left portion of junction) and (b) ∼5% of the area (lower middle portion of junction). The animals were allowed to recover from surgery and then re-anesthetized the next day and the neuromuscular junctions relocated. In each case, the small remaining input grew to occupy the entire neuromuscular junction at some time prior to 24 h. In addition in both cases the caliber of the axon entering the junction enlarged. The axons were imaged in singly and doubly transgenic mice that had constitutive cytoplasmic expression of (A) YFP (green) and (B) both CFP (pseudo-colored green) and YFP (red), respectively. In the latter there was a greater proportion of CFP in the smaller axon, making it appear greener (in the first panel) and thus possible to distinguish its territory from the sites occupied by the other axon even before laser axotomy. Scale bar, 20 µm.

Damage to axons typically evolved over 30–45 min and the whole process of axon removal required many hours. Even though we observed bleaching of the axon segment at the time of irradiation, evidence for structural damage only became apparent within 10–20 min (see Figures 1–3). Signs of axon damage included dramatic swelling of the axon distal to the site of laser focus and a progressive widening of the region of non-fluorescence both distal and proximal to the laser irradiation site. Presumably this loss of fluorescence is secondary to leakage of proteins from the cytoplasm at the damage site. This phase which typically lasted up to several hours was followed by the complete disappearance of the distal axon save for occasionally a few small disconnected fluorescent fragments that ultimately all disappeared by 10 h. In the proximal direction the damage initiated a die-back that was reminiscent both in time course and scale of “acute axonal degeneration” of damaged central axons [26]. Typically, the die-back stopped at the proximal branch point (Figure 2), although sometimes it extended anterogradely from the branch point to cause the disappearance of other terminal branches. If the fluorescence at the laser spot recovered after several minutes, that was an indication that the fluorescence in the axonal branch had been bleached but the axon was not seriously damaged because no subsequent changes were noted over the next half hour to hour, or the following day (see Materials and Methods for details).

### Laser Removal of One Axonal Input to a Multiply Innervated Neuromuscular Junction Invariably Leads to Takeover of the Synaptic Site by the Remaining Input

We attempted to remove one of the axons converging at multiply innervated neuromuscular junction in early postnatal life. In anesthetized mice that were 7–8 d old we located neuromuscular junctions in the superficial (ventral) surface of the sternomastoid muscle that were innervated by two axons. In the sternomastoid muscle about half the neuromuscular junctions are multiply innervated at 1 wk of age, whereas at 2 wk the number of multiply innervated neuromuscular junctions is very small (<0.1%) [8]. At 1 wk nearly all of the multiply innervated junctions are contacted by only two axons [4],[7], indicating that each of these junctions will lose one input over the next several days. Using multi-photon laser irradiation we successfully removed one axon from each of 15 multiply innervated neuromuscular junctions. At an additional 87 neonatal muscle fibers, axons were damaged but the experiment failed for other reasons including connective tissue buildup and muscle fiber rotation that obscured details when we returned to the muscle the next day; inadvertent muscle, nerve, or blood vessel damage; or occasional animal mortality post-surgery. After confirming that the axon was damaged during the imaging session (up to 3 h) we sutured the neck wound and allowed the animals to recover. In most cases (10/15) we intentionally irradiated the axon with the larger caliber. In 4/15 junctions the two axons had nearly the same caliber, but based on their appearance at the site of entry into the junction, we could identify the axon that we thought had less territory; we then irradiated the larger input. In the remaining case we intentionally laser irradiated the axon with the smaller caliber. In all of these junctions the selectivity of the laser damage was apparent because whereas the damaged axon disappeared, in no case did the non-targeted axon show any swelling, fragmentation, bleaching, or loss (Figures 1–3). Because in this experiment the two axons were labeled with the same fluorescent protein and in most cases their territories coalesced at light microscopic resolutions, it was not possible to know precisely how much territory the remaining axon occupied except retrospectively. Once the laser irradiated axon had disappeared, it was easy to see the extent of the territory occupied by the remaining axon (Figure 3B). In the 14 cases where we attempted to remove the stronger input, the remaining axon occupied half or less of the junctional acetylcholine receptor (AChR) sites (mean 12%), and in the one case where we irradiated the thinner axon, the remaining axon occupied 80% of junction (Table 1). The territories occupied by these axons were consistent with previous work showing that terminal axon caliber correlated with synaptic territory [7]. Because the axon occupying the majority of the territory at postnatal day 7 or 8 (P7 or P8) was more than twice as likely to remain at a junction than the axon occupying the minority of the territory [4], we would anticipate that in most of the 14 cases where we targeted the stronger axon, the remaining undamaged axon would have been eliminated had we not perturbed the system.

**Table 1:** Synaptic takeover at developing neuromuscular junctions following laser-induced damage of an axonal input.

(A) Dually Innervated Junctions				(B) Junctions with Retracting Axon Nearby			
% Occupancy		Axon Diameter (µm)		% Occupancy			Distance from NMJ (µm)
0 h	+1 d	Undamaged	Ablated	0 h	+1 d	+2 d	
80	100	2	1.4**	0	0	1	14.4
50	100	1.2	1.2	0	1	100	9.6
30	75	1	1.4	0	10*	—	2.4
20	100	1.2	3	0	10	—	33
10	50*	1	1.8	0	10	—	29.8
10	100	1	1	0	40	100	6
10	100	1	1.8	0	50	—	2
10	100	1.2	1.4	0	90	100	9
5	100	1	1.4	0	100	—	4.4
5	100	1	1.8	0	100	100	3
5	100	1.4	1.4	0	0	—	1.8
5	100	0.8	1.4	0	0	—	10.6
5	100	0.8	2	0	0	—	11
1	100	2.2	2.2	0	0	—	14
1	100	1	1.4	0	0	—	15.8
				0	0	—	16.2
				0	0	—	19.4
				0	0	—	63.2

*: t = 17 h. (A) At 0 h the larger diameter (“Thick”) input was removed by laser illumination. In one case (**) the smaller diameter (“Thin”) input was removed. Axon caliber was correlated with territory occupied at the junction. In general the “Thin” input occupied less territory than the “Thick” input. The territory occupied by the remaining input (% occupancy at 0 h) was measured relative to the territory occupied by the two together (100%). In every case the remaining input grew and took over the synapse. In most cases (*n* = 14/15) takeover was complete (100% occupancy) by 24 h. Takeover occurred even when the input was very small (1%–5% occupancy at 0 h). (B) Synaptic takeover also occurred at junctions that were singly innervated and had a retracting axon nearby, that is, where synapse elimination had recently been completed. In this case, one input occupied 100% of the junction, and the other occupied 0% at the time of laser irradiation. In these cases, removal of the innervating input often resulted in the retracting axon growing back to occupy the junction (55%, *n* = 10/18). In the remaining cases the retracting axon did not grow back and/or had disappeared when the junction area was viewed the next day. The distance of retracted axons from the junctions at the first time point (0 h) is indicated in the far right column.

Nonetheless, when we re-anesthetized the mice a day later and returned to the same muscle fibers, in none of the 14 cases had the remaining axon withdrawn. Nor in any case did we see any evidence of regrowth of the damaged axon. We were certain that the axon remaining at the junction was the axon that was not irradiated on the previous day because its site of entry into the junction was in each case the same as the site where the thin axon was situated on the day of laser irradiation (see Figure 3).

In each case, however, the axon that remained changed in a striking way. Each of these thin axons now extended branches throughout the postsynaptic area to fully occupy the territory formerly overlain by the laser irradiated axon. In all but one case the growth response appeared complete within the first 24 h after laser exposure. The axonal expansion of territory was reminiscent of the “takeover” seen during normal synapse elimination at the neuromuscular junction: the advancing axon specifically enlarged its coverage of the adjacent postsynaptic AChR sites without extending sprouts to new sites [4]. Because ordinarily smaller axons were twice as likely to leave a multiply innervated junction than larger ones [4], the probability that none of them (14 cases) would have withdrawn is very low (probability <0.0000003). Therefore, the fate of the weaker axon was changed by removing the stronger input, a result that argues that competition is the cause of the elimination of the weaker input.

We were interested to know what occurred in the immediate aftermath of removing the other input. In particular, did the remaining axon continue to be eliminated for some time, suggesting, for example, that competitive effects have some momentum and a certain amount of time is required before an axon can change its fate? We found, however, that at no time after axon removal did the remaining axon show any evidence of continued elimination. In three cases we reimaged junctions less than 24 h after the initial view (one at 6 h, one at 12 h, and one at 17 h). At the 6 h and 12 h views, the remaining axons had not lost any territory (see Figure 2). The junction viewed at 17 h had already shown signs of expansion. Unfortunately, it was not possible for us to anesthetize the same animal for imaging more than once per day and have it survive, so the exact time axons began to grow following laser damage of the other synaptic occupant remains unclear.

We do know, however, that at some point after a period of quiescence that lasted up to 12 h, the territory occupied by the remaining axon changed rapidly. In 13/13 junctions imaged at 24 h after laser induced removal of the stronger input, the remaining axon had expanded dramatically. In all but one case the axon occupied the entire postsynaptic site, and in the one other case, it occupied 75% of it (Table 1A, Figures 1 and 3). The increase in territory by the remaining axon was often matched by a thickening of the caliber of its preterminal branch (Table 1A, Figures 1 and 3). In the one junction we studied in which the weaker input was intentionally eliminated, we also noted complete takeover of its territory by the stronger axon at 24 h (Table 1A). Thus by 1 d after the laser removal of one axon at dually innervated neuromuscular junctions, the remaining input had grown and now appeared identical to the axons that survive naturally occurring synapse elimination and singly innervate neuromuscular junctions. In each case the undamaged axon now occupied all or nearly all the postsynaptic territory and possessed a thick preterminal axon. Importantly, in 6 of the 15 cases, the axon that remained had occupied less than 5% of the junction at the time of axon removal. These axons were as effective in taking over the remaining territory as axons that had a larger footprint at the time of axon removal (Figure 3B and Table 1A). We thus conclude that once a competing axon is removed the remaining axon, within hours, and irrespective of the contact area of its terminal arbor, changes its fate to take the position and characteristics of the dominant axon.

### Retraction Bulbs Can Quickly Change Fate, Reverse Direction, and Reinnervate Neuromuscular Junctions

**Figure 4:**
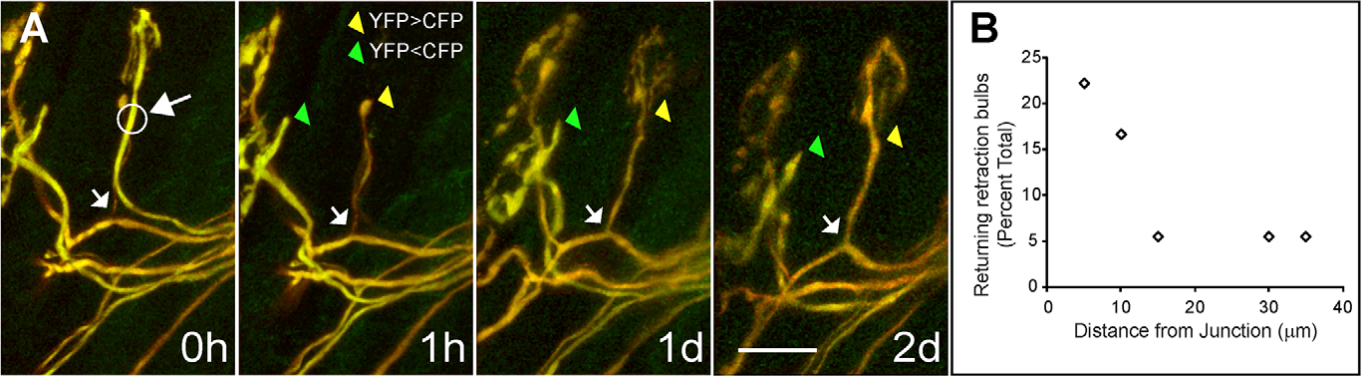
Previously retracted axons reinnervate their former neuromuscular junctions after laser-targeted removal of the innervating axon. (A) Shown is a singly innervated neuromuscular junction with a nearby recently retracted axon (tipped by a bulb) in the sternomastoid muscle of P7 mouse in vivo. The pulsed laser irradiated the axon innervating the neuromuscular junction at the circle marked by a large arrow. After 1 h, the irradiated axon was mostly invisible, leaving the bulb-tipped retracting input unchanged. By the next day (1 d), the retracting input had reversed direction and nearly completely reinnervated the junction. After 2 d (2 d) the caliber of the reinnervating axon and its terminal branches increased. In this experiment it was unambiguous that the regenerated axon was the former withdrawing axon because the two axons could be distinguished by their relative concentrations of YFP and CFP. The bulb-tipped axon could also be re-identified after it reinnervated the junction because of the location of its proximal branch point (indicated by the small arrow in each image). Scale bar, 20 µm. (B) Graph showing the incidence of return of retracting axons as a function of its distance from the junction at the time of laser axotomy of the innervating axon (also see Table 1B). The percentages were computed relative to the total number of retracting axons studied. Retracting axons 10 µm or closer appeared more likely to return than ones farther away.

Once an axon has lost all territory at a neuromuscular junction it undergoes a stereotyped process of withdrawal in which the bulb tipped axon branch sheds some of its cytoplasm and appears to retract away from the junction [11],[18],[27]. These “retraction bulbs” are seen frequently in developing muscles at the time of synapse elimination but are not seen at all in adult muscles. Might these structures be irreversibly committed to retraction? In anesthetized mice we successfully damaged 18 strong axonal inputs of singly innervated junctions where a second axon had recently retracted but was still visible nearby. Previous time lapse studies indicate that retracted axons which were within ∼200 µm of a junction had disconnected at some point over the previous 48 h [4],[11]. After damaging the axonal input that innervated the junction, we allowed the animals to recover and waited to see if nearby retracted inputs ever attempted to return to the junction over the following days. To our surprise, in 55% of the cases (*n* = 10/18), the axon stopped retracting, grew back to the junction, and occupied the entire junctional area (Table 1B). The laser-irradiated axon typically died back to the proximal branch point. Although in most cases given the position of the two axons it was unambiguous that the damaged axon did not reinnervate the junction, a potential ambiguity could occur if the damaged axon rapidly reinnervated the junction while at the same time the retracting axon completely disappeared. In order to directly identify the re-growing axon, we used a doubly transgenic mouse in which individual neurons expressed different concentrations of cyan fluorescent protein (CFP) and yellow fluorescent protein (YFP) in each axon (see Materials and Methods). In this case, we found that the damaged axon (identified by its color) did not return over the next 48 h, whereas the retracted axon (unambiguously identified by its color and its site of exit from the nerve fascicle) reinnervated the junction within 24 h and increased in caliber over the next day (Figure 4A).

In order to determine why some retracting axons succeeded in regrowth whereas others did not, we compared the fates of retracting axons at various distances from their previous neuromuscular junction. In three of the cases, we infer that the retracting axons had only recently been eliminated because at the time of laser irradiation of the dominant axon they still had a fine filamentous process connecting them to the junctional site. All of these retracting axons reoccupied the junction 24 h later. But such a tendril was not required for reinnervation: 7/16 of the remaining retracting axons also reoccupied the junctions despite not being connected. Generally, we found that retracting axons close to the junction were significantly more likely to grow back than ones farther away (Figure 4B). These results indicate that once an axon has disconnected from a junction, there still may be a window of 1–2 d before it has transitioned to a mode where retraction is irreversible. In summary, the ability of retracted axons to return to a junction suggests that growth stimulating signals and/or signals that disinhibit the retraction process are activated following laser axotomy. The delay in initiation of growth appears to be nearly the same whether or not the undamaged input was in direct contact with the laser damaged axons at the junction, suggesting that the onset of growth response is not a result of the loss of contact inhibition. It is, however, clear that the time required to completely reinnervate a neuromuscular junction is a bit slower for axons that have completely disconnected and have a longer distance to grow (see Table 1B).

### Withdrawal Before Takeover During Naturally Occurring Synapse Elimination

**Figure 5:**
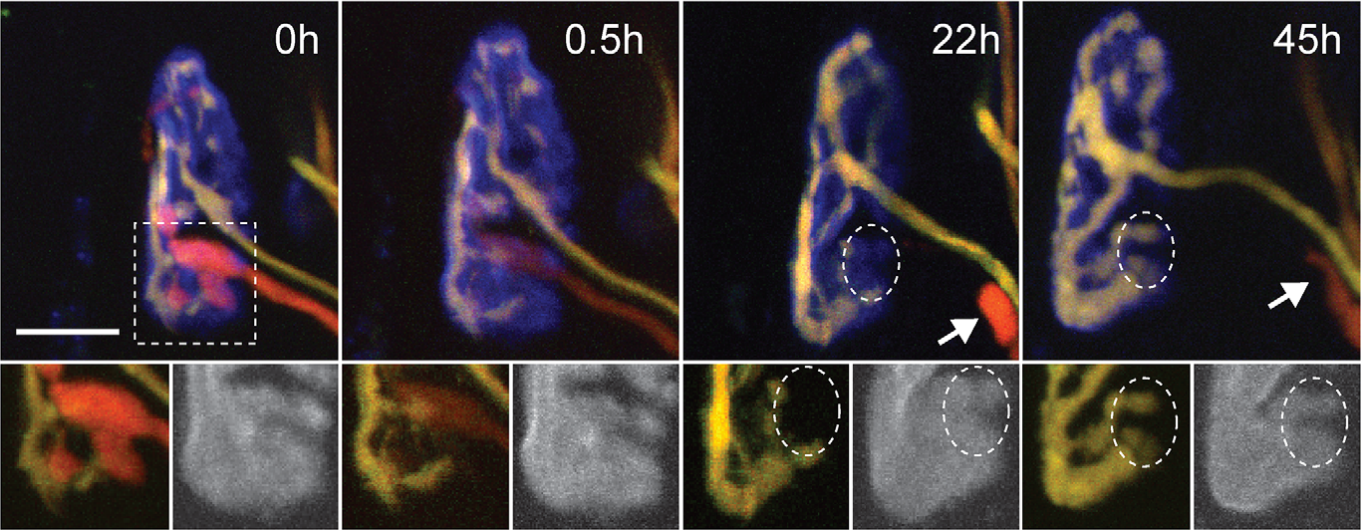
Synaptic withdrawal precedes takeover during naturally occurring synapse elimination. Shown are four views of a multiply innervated neuromuscular junction from the sternomastoid muscle of a living mouse viewed over 2 d starting at P10. AChRs were lightly labeled with fluorescently tagged α-bungarotoxin (pseudo-colored blue). Both axonal inputs contain CFP (pseudo-colored green) and YFP (red) but in different proportions, allowing one input (red+green = yellow) to be distinguished from the other (orange). At the first view (0 h) the orange-colored axon occupied the lower part of the junction and a small branch at the top. Insets below show axons (left) and fluorescently tagged AChRs (right, in gray) of the bottom region of the junction. To more clearly see the extent of the territory occupied by the yellow axon, the fluorescent protein in orange-colored input was bleached at the nerve entry zone for several minutes without damaging the axon (see Figure S2) using visible continuous wave laser light. A day later (22 h) the orange-colored input is no longer at the junction, but a remnant of the retracting axon is visible (arrow). Several sites that had previously been occupied by the orange axon are now vacant (dashed ellipse). However, by 45 h, the remaining input grew into the vacated territory. The delay between the withdrawal of the orange axon and the takeover by the yellow one is similar to time course observed following removal of an input by laser irradiation (approximately 1 d). Scale bar, 10 µm.

The results described above show that axons that are in the midst of withdrawing from a synapse can be stimulated to grow and occupy the recently vacated sites of a damaged axonal competitor. We were interested in learning if this method of axonal takeover also underlies the way synaptic competition occurs in normal development. In particular there are two alternative ways axons might enlarge their territory. One way is that an axon expands its territory by “pushing off” a competing axon. If this were the case, each AChR region is sequentially occupied first by one axon and then by another with no lag in takeover and no interval when the AChRs are unoccupied. An alternative way an axon might enlarge its territory is if the stimulus for an axon to take over a site is generated in response to that site's vacancy following withdrawal of another axon. This latter mechanism is what likely stimulates the growth of axon terminals following laser axotomy of a competing axon. Previous studies did not have the necessary spatial resolution to resolve this question at sites of synaptic takeover; however, in one situation where the two axons' territories were widely separated, sites that were vacated by one axon were not taken over by the other. That result rules out the idea that in all cases synapse elimination requires one axon to displace another from a synaptic site [4]. In this work we wanted to extend our analysis to sites of takeover. We reasoned that if synaptic vacancy were the proximate cause for synaptic takeover in naturally occurring synapse elimination, then it should be possible to observe instances of transiently vacated AChR sites at the boundaries of the territories occupied by different inputs. Whereas if one axon only lost its territory when another invaded its site, then no lag should ever be seen. In two neuromuscular junctions we did observe synaptic territories that were unoccupied at the first imaging session and then occupied a day later. The presence of unoccupied receptors suggests but does not prove that one axon had withdrawn and the other axon was responding by taking over those sites. More direct evidence would require seeing one axon withdraw from a site that would then appear vacant before another axon would later reoccupy it. Our previous experience suggested that finding examples, were they to exist, would be difficult because in most cases competing axons overlap in some regions with one sitting on top of the other, meaning that if withdrawal precedes takeover, the axon branches are likely lifting but not retracting before takeover occurs. The distances involved are below the resolution limit of the microscope, making it difficult to know if AChRs are transiently unoccupied or not [12]. In addition, we know that takeover following laser axotomy occurs rapidly, but previous time lapse images of retraction suggest that removal of a detached branch occurs more slowly, meaning that the takeover may be happening before the other branch disappears [4]. We thus devised a photo-bleach method to unambiguously see the extent of overlap at neuromuscular junctions as a way to screen for multiple innervated junctions where the two axons abutted each other but had minimal overlap (see Materials and Methods). Such junctions would be most likely to reveal the transient presence of a vacant AChR site. In one case out of hundreds of attempts we did see the withdrawal of one axon leading to AChR vacancy and then the takeover of that site by a second axon (Figure 5) over the course of 3 d. In the first view the two inputs appear to overlap in some areas but not at the site of axon entry. After 1 d, the smaller input has withdrawn completely from the junction. A vacated AChR region is seen at the site of axon entry. By the following day, this region has become occupied by the remaining input. It is clear in this example that withdrawal preceded takeover and that the takeover occurred after a delay of many hours. The timing of takeover is similar to what we found following removal of an input by laser irradiation. Because we did not observe takeover in the first hours after removal of an axonal input by laser irradiation, we think it is likely that takeover is stimulated by the withdrawal (i.e., vacated sites) and that withdrawal before takeover is possibly the general mechanism by which synaptic contacts are rearranged at junctions. However, based on some of the data in this article (see section below) and previous studies [4], it seems likely that a vacant site is a necessary prerequisite for axonal growth but not a sufficient stimulus. For example, when a vacant site is nearby other innervated sites, it is very likely to be reoccupied. But when the site is off by itself at the edge of a junction or at the nerve entry site, then the vacant site is sometimes not reinnervated and the receptors eventually disappear.

### Axon Growth Induced by Small Unoccupied Synaptic Sites within Innervated Neuromuscular Junctions

**Figure 6:**
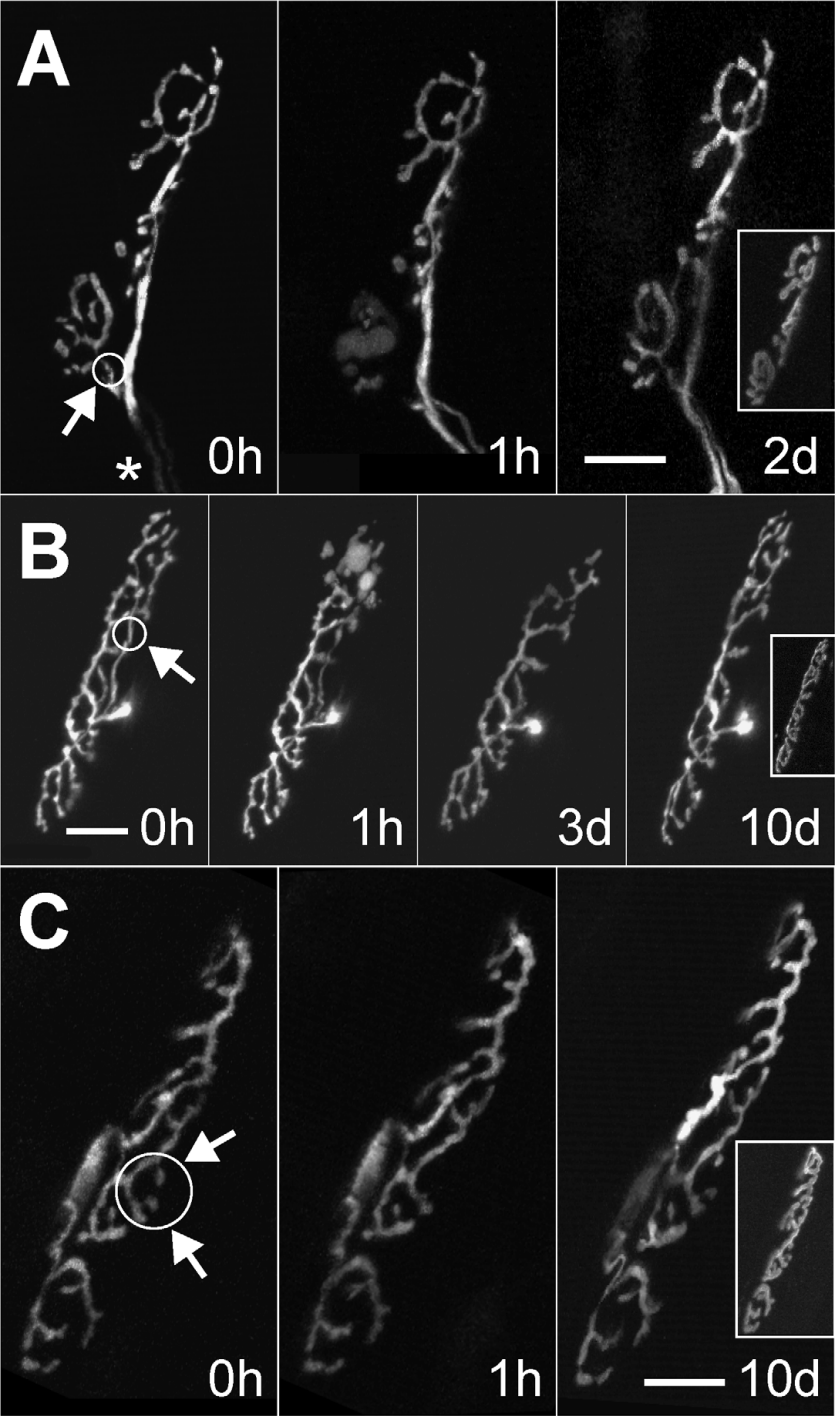
Small synaptic vacancies within innervated neuromuscular junctions induce axon growth. Singly innervated neuromuscular junctions were imaged in vivo in the sternomastoid muscle of adult transgenic mice expressing green fluorescent protein (GFP) in all motor neurons. After pulsed laser-targeted irradiation (circles and arrows indicate sites of laser-induced damage) the nerve terminals were re-imaged at 1 h to confirm damage to the targeted branches. The size of the denervated synaptic area relative to the total area of the junction was approximately (A) 30%, (B) 10%, and (C) less than 5%. AChRs of each neuromuscular junction are shown in the insets of the rightmost panels. The same nerve terminals were imaged again 2–10 d later. In (A) two branches of the same axon innervate neighboring regions of the neuromuscular junction (asterisk). Reinnervation of the bottom portion was complete within 2 d of laser irradiation. In (B) an axon branch that innervated the upper part of the junction was irradiated. Reinnervation of the unoccupied receptor sites was complete at some point between 3 d and 10 d after laser axotomy. In (C) the terminal boutons overlying two small synaptic sites were removed via laser irradiation and then became reinnervated. Given the large safety factor for muscle activation it is unlikely that the small regions that were denervated had any effect on muscle fiber activity; nonetheless, the nerve grew to reoccupy these sites. Scale bars, 20 µm.

The results above suggest that a signal from a vacant site may stimulate the growth response during naturally occurring synapse elimination. In many situations axonal growth is thought to be stimulated by signals that emanate from denervated, and thus inactive, target cells [28]. However, in the cases of normal takeover in development and in the case where we targeted the laser to a weak input, the loss of an axon was unlikely to give rise to target inactivity because we had denervated a minuscule portion of a large synapse. We were thus interested to know if small partial denervations of target cells are generally sufficient to activate an axonal regeneration response. In particular if only a small synaptic bouton is removed and the muscle fiber is not functionally denervated, will sprouting be stimulated? As described above we found one case in development where a small site became unoccupied and then later reoccupied by the remaining axon (see Figure 5), but we wanted to see if this was a general trend that occurred if vacant sites were present at any age. We were also interested to know whether the proximate cause for the sprouting within a neuromuscular junction could be explained by release of contact inhibition. We thus did focal laser axotomies in adult neuromuscular junctions to denervate small isolated synaptic boutons while retaining innervation to the rest of the junction. Surprisingly in adult animals more than half (55%, *n* = 12/22) of these small laser-targeted axonal surgeries which denervated between 5% and 30% of the AChR sites still induced reinnervation (Figure 6). The reinnervation started after a delay of at least 1 d following laser exposure and was typically complete by 2 d but sometimes longer (see Figure 6B). In all cases reinnervation occurred by sprouting from adjacent branches in the terminal. The sprouts appeared to be directed specifically to unoccupied AChR sites and not elsewhere, yet the original branching pattern (pre-irradiation) was not necessarily preserved, suggesting that regrowth was not necessarily guided by preexisting glial sheaths but by highly localized signaling originating at the vacated sites.

## Discussion

This study was undertaken to better understand the sequence of events that occur during development underlying the transition from multiple to single innervation in skeletal muscle. This phenomenon, which has analogs in other parts of the developing nervous system, occurs by one axon's takeover of most of the postsynaptic sites that were earlier occupied by other axons. However, a number of questions about the underlying mechanism remain unanswered. First, what drives the exchange of territory such that when one axon loses sites another typically gains those sites [4]? Second, what determines the identity of the eventual surviving input given that an axon that loses territory at one time point sometimes gains it back at a later time [4]? And third, why do the contacts of an axon within a neuromuscular junction tend over time to cluster to occupy a contiguous segregated territory [29]? In this work we focused on answering the first question. In so doing we think we have also uncovered explanations for the other questions and believe we now have a framework to interpret many aspects of this form of synaptic plasticity.

We show that axons rapidly respond to vacant synaptic sites by growth. In multiply innervated neuromuscular junctions an axon whose elimination appears imminent will, within 1 d, occupy all the sites of an axon that was experimentally removed. Moreover, axons that have recently withdrawn completely from a neuromuscular junction will reverse their fate and reoccupy it if the innervating axon is caused to disappear. These results strongly support the idea that the process leading to single innervation is competitive: an axon destined for elimination always survives if the other innervating axon is removed.

This growth response of one terminal axon branch to the damage of another terminal branch is in some ways reminiscent of the reinnervation response following partial denervation of a muscle where an axon that is undamaged sprouts to occupy neuromuscular junctions on denervated muscle fibers [30]. However, these two phenomena seem to be dissimilar in several important respects and may have different underlying mechanisms. First, a number of studies support the idea that sprouting following partial denervation is stimulated by muscle fiber inactivity [30],[31]; however, several of our results show axons growing into vacated synaptic sites even when the muscle fiber is functionally innervated. It is also clear that in naturally occurring synapse elimination, an axon continues to take over vacated sites even when it already occupies the vast majority of the terminal area so that its growth is not being stimulated by inactivity of the muscle [4]. A second difference between partial denervation of muscle and the growth response described here is that the following partial denervation axons grow through vacated Schwann cell tubes [32] or extend along new Schwann cell processes [33]. Neither of these paths is available within neuromuscular junctions. Third, the growth response following laser axotomy in neonatal animals is fast compared to the response of axons in adults to partial denervation. Another difference is that many of the axons undergoing branch loss in development were atrophic and had to transition from a withdrawing state to a growing state, whereas the axons responding by growth following partial denervation in adults are in a healthy quiescent state before being induced to grow. These differences suggest that the local growth response to synaptic vacancy within a neuromuscular junction is different from the growth response of axons to the loss of all innervation to a subset of muscle fibers (i.e., partial denervation).

Because muscle fiber inactivity is unlikely to be the stimulus that induces axonal growth into vacant synaptic sites in our studies, what then is the signal? One idea is that Schwann cell processes that no longer are associated with an axon become activated. Previous studies have shown that Schwann cell activation following nerve damage is a potent stimulus for axon growth [34],[35]. Thus, it is possible that focal loss of nerve-glial contact leads to the release of a glial-derived signal that causes axons to grow. Interestingly glial cell-derived neurotrophic factor (GDNF), a glial based growth factor, is one of the strongest known stimuli for mammalian motor nerve growth [36],[37]. Growth based on loss of nerve-glial contact is an attractive idea because it does not require muscle inactivity as a stimulus. Such a mechanism could be the same as the one that promotes axon regrowth along nerveless Schwann cell tubes to distant muscle targets following nerve damage far from muscle end organs (such as the sciatic nerve) [38]. Another possibility is that Schwann cell activation is downstream of a signal originating in the postsynaptic cell or that the vacant postsynaptic site signals axon growth directly. Ongoing experiments are aimed at deciding between these alternatives.

The results presented here suggest the primacy of the withdrawal (or loss of maintenance of synaptic contacts) as the initiating event leading to synaptic takeover and ultimately single innervation of neuromuscular junctions. Interestingly, signals from presynaptic, postsynaptic, and glial cells all seem to be able to regulate synaptic maintenance. For example, at the mammalian neuromuscular junction, synaptic loss can be initiated by postsynaptic protein synthesis inhibition [10], focal blockade of neurotransmission at a synaptic site [8], exuberant branching of motor axons in development [13], terminal sprouting [35], and glial loss [39],[40]. A molecular understanding of developmental synaptic reorganization may therefore require understanding the relative roles of these diverse signals and cells in causing synapse loss. The important point from our perspective is that by focusing on synapse loss (as opposed to synaptic addition) it may be possible to get to root causes for the rearrangements.

**Figure 7:**
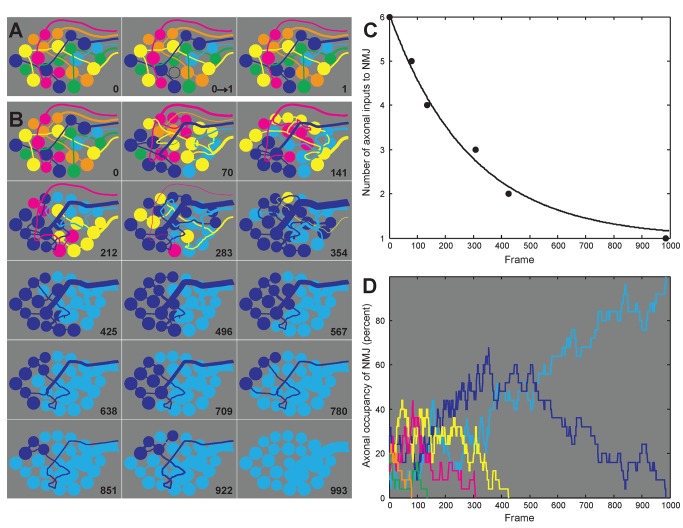
Model of synaptic competition based on piecewise withdrawal from synaptic sites. Inspired by models of population dynamics of species competing to occupy the same niche, we produced a simple graphical model of neuromuscular synaptic competition that gives rise to many of the features that have previously been observed (see Materials and Methods for model details). (A) We represent a junction by a set of synaptic sites that, in this simulation, are randomly distributed among six innervating axons each with a different color (see left panel). Based on the findings in this article showing that synaptic vacancies can induce nearby axonal branches of either the same or a different axon to grow, we simulated synaptic competition as an iterative process of axon withdrawal from a randomly selected site followed by takeover of the vacated site (see also Video S1). As shown in the first two panels the process starts with the pink axon losing a synaptic contact. The vacated site is subsequently reoccupied by one of the immediately neighboring axons (in this case the dark blue one; see right panel). (B) This stepwise process is repeated approximately 1,000 times until all the sites become innervated by the same axon and the junction is in its mature singly innervated state. Over the course of this simulation, the synaptic contacts of each axon become progressively clustered, and eventually, when the junction has only two remaining inputs, the axons become completely segregated as has been seen in normal development [29]. (C) Graph showing that in this model there is a rapid loss of inputs in the first postnatal week (when many axons converge on each junction) compared to the second postnatal week (when junctions are innervated by at most two axons). This result is consistent with recent experimental observations [45]. (D) The model also shows that an axon's territory does not necessarily change in a monotonic way and that the final outcome cannot be predicted with certainty by the relative amounts of territories that each axon occupies. Notice, for example, that the axon with the least amount of territory at time 0 (the light blue axon) is the ultimate winner. These results are consistent with the “flip flop” described and observed in vivo [4].

Based on these results we developed a surprisingly robust graphical model simulating synaptic competition (Figure 7). In this model we posit that maintenance of synaptic contacts is imperfect and that at regular intervals an axon withdraws from an individual synaptic site at random. However, in each case the consequence of the vacated site is the same: as shown in this work, when an axon withdraws from a synaptic site, signals stimulate nearby axons to grow and attempt to occupy that site. In this model the reoccupation favors axons that have the largest number of nearby synaptic contacts (Figure 7A and see Materials and Methods for details). This proposed mechanism is inspired by models used in evolutionary biology for understanding the survival and extinction of different populations within the same niche [41]. In this case the different populations that are in competition are the several axons converging on the same neuromuscular junction, with each synaptic contact being equivalent to an individual member of one or another population. As shown this process will lead eventually to single innervation (Figure 7B). This simple model also provides insight into the cause of several other features of synaptic competition such as synaptic segregation [29], flip-flop [4], and the slowing pace of input loss in the second compared to the first postnatal week (Figure 7B and C). Thus, loss-initiated synaptic takeover suggests a useful conceptual framework for understanding competitive synaptic rearrangements at the neuromuscular junction.

An important question is whether the model proposed here also has relevance to synaptic rearrangements occurring in other parts of the nervous system. It is well known that axons are lost from neurons at the same developmental stage that axons are removed from muscle fibers. In several cases it is also clear that a remaining input to a neuron (such as the climbing fiber on a Purkinje cell or a preganglionic axon on a submandibular ganglion cell) elaborates new synaptic connections at the time other axons are being eliminated [42]. The complementary nature of the loss and gain of connections by withdrawing and remaining inputs, respectively, raises the possibility that a remaining input is induced to grow and innervate synaptic sites that have become vacant because of axon loss. In the climbing fiber system, the final area occupied by the surviving axon is far greater than the area occupied by the multiple axons initially innervating the Purkinje cell soma. This large increase in area is a consequence of the Purkinje cell elaborating its dendritic tree at the same time climbing fiber axons are being lost from the soma [43]. In this case new (and hence vacant) postsynaptic sites on the expanding dendritic arbor may stimulate the climbing fiber to “climb” from the soma and grow out along the dendrites. In autonomic ganglia it is also clear that axons elaborate synapses on the soma and then grow out to innervate sites on the dendrites [44]. Thus in both of these cases rearrangement of synaptic connections has two parts: (1) loss of some axonal inputs and (2) concomitant elaboration of additional connections by the axons that survive. Hence the mechanism by which motor axons grow locally in response to vacated sites at the neuromuscular junction may inform on and be analogous to the mechanisms underlying the establishment of both the correct number of innervating axons and their total synaptic drive on neurons (see also discussion in Tapia et al. [45] elaborating the idea set out above that the mechanism for competitive synaptic rearrangements is consistent with a net increase in synaptic numbers and an important general role for elimination during neural development). Moreover, if our evidence of continuing plasticity in adult muscles is any guide (see Figure 6), a kind of local structural plasticity might continue on multiply innervated neurons even in the adult brain. As such “evolution” of neural network connectivity from a dynamic competitive state to one marked by long-term functional stability could be the basis of indelible alterations in brain function such that occur with learning.

## Materials and Methods

### Transgenic Mice

Mice that expressed either cytoplasmic GFP (line GFP-I^+/+^), YFP (line YFP-16^+/+^), or both CFP and YFP (lines CFP-5^+/+^ or CFP-23^+/+^ crossbred with YFP-16^+/+^) were used for all experiments (protocols approved by Washington University Animal Studies Committee and Harvard University's Institutional Animal Care and Use Committee) [46]. The GFP line was used for studies in adult mice. The YFP and CFP lines were used for neonatal mice. The GFP line could not be used for imaging in neonatal mice because the onset of GFP expression in motor neurons was delayed until after the first postnatal week.

### Multicolor Axon Labeling and Photobleaching

To be able to visually distinguish one input from another we developed a method of labeling axons in multiple colors using very bright thy1-driven XFP transgenic mouse lines. By crossing two lines in which all motor neurons constitutively express a fluorescent protein, we found that in young animals intrinsic variation in expression level of each fluorescent protein between neurons was sufficient for us to distinguish axons by color. An advantage of this approach was that every multiply innervated junction on the muscle surface was a potential candidate for imaging. A challenge was that the spectral separation of the axons was incomplete when imaged using conventional confocal microscopy. Because every neuron expressed the same two fluorescent markers (varying only in linear combination), all the axons appeared in both fluorescence channels. Thus fluorescence of one input obscured the fluorescence of another where there was overlap between the inputs. To achieve complete color separation, we found that we could temporarily and selectively photobleach one or both fluorescent proteins in an input, changing its color and eliminating its fluorescence completely for a time (∼minutes). This photobleaching was accomplished using the standard confocal lasers that were using many orders of magnitude less power than the IR irradiation for laser axotomy. A region of interest (ROI) in the scanning confocal was located where we could illuminate the terminal of a single axonal input. We used the color differences between axons at their entry point to identify where to focus the laser at the terminal for photobleaching one axon without bleaching the other. The ROI was scanned by zooming the laser scanning confocal to cover that area but not the other axon. We followed the effectiveness of the bleaching by visualization of the decrease in fluorescence emission. CFP or YFP were bleached using 440 nm or 514 nm light, respectively, at 100% power for 2–5 min. The animal ventilator was left on while bleaching. An image stack of the neuromuscular junction was then acquired immediately after bleaching to capture the change in that axon's color before fluorescence recovery. We found that because the fluorescence recovers quickly, presumably by dilution with unbleached fluorescent protein in the more proximal axon, the same region could be photobleached repeatedly and without any apparent phototoxic effects (see [47] for control experiments). We assume that because the fluorescent proteins were freely diffusible in the cytoplasm and not tethered to any important organelles or membranes, we noted no immediate (minutes to hours) or delayed (days) toxic effects on the irradiated neuron (see Figure S2). Once bleached, the areas where the two axons overlapped in the junction were disambiguated by taking images of the junction with each laser line.

### Imaging

Adult and neonatal mice were anesthetized, intubated, and respirated as previously described [4],[47],[48]. The ventral neck skin was incised and retracted laterally to expose the right sternomastoid muscle, which was gently lifted on a flat steel platform. Care was taken not to stretch the muscle in order to prevent damage to the muscle, blood supply, or innervating nerve bundle. The wound was filled with sterile saline and a glass coverslip was placed over the muscle, making a meniscus with the saline to help keep the muscle from drying. The coverslip did not touch the muscle. Axons innervating the central band of neuromuscular junctions were visualized using standard epifluorescence optics (YFP filter cube) at high magnification (60× 0.9NA water immersion objective). Once a particular nerve terminal was selected, images were taken using a confocal microscope (Bio-Rad MRC1024MP or Olympus FV-1000). GFP-filled axons in adult animals were illuminated using 488 nm light. In neonates, YFP-filled axons were illuminated using either 488 nm light or 514 nm light, and axons that were filled with both CFP and YFP were illuminated using 458 nm and 514 nm light simultaneously. The ventilator was turned off temporarily (30–60 s) to acquire a stack of images. Axons and acetylcholine receptors were imaged sequentially. To avoid damage to the muscle and synapse, acetylcholine receptors were labeled only after laser ablation of axons. Receptors were lightly labeled with alexa-647 conjugated α-bungarotoxin (5 µg/ml in PBS for 40 s), and the muscle was then rinsed well with PBS. This dosage did not paralyze the muscle, as greater than 70% of the receptors remained unlabeled [4]. Movement artifacts were removed from stacks using special alignment software (Autoquant; Media Cybernetics, Inc). A 2-D image of each junction was then obtained by a maximum intensity projection. The same junction was imaged multiple times, before and after laser ablation, and in most cases at 1-d intervals thereafter. The mice were resuscitated after each imaging session as previously described [4],[47]. In the time lapse views the color balance of the image from different time points was sometimes corrected using the background autofluorescence as a reference.

### Laser Ablation

Laser ablation was performed using a Spectra Physics Tsunami Ti:S laser oscillator and pumped by a Millenia 5W solid state laser using scanning mirrors from a laser scanning microscope. The pulsed laser output was tuned to 815 nm. The laser power was approximately 120 mW at the back aperture of the objective. An IR-corrected water dipping cone objective (LUMPlan W-IR2 60× 0.9NA Olympus) was used to focus the laser onto an axon branch. The scanner's mirrors were parked (i.e., maximal zoom) to position the laser onto a ∼0.5 µm spot (diffraction limited). In this way all of the laser's power was focused to the waist of an hourglass-shaped beam giving a football-shaped spot (long axis ∼2.4 µm). The damage required 30 s to 1 min of mode-locked operation of the laser (approximately 1nJ energy per 80 fs pulse). This laser axotomy approach differs from a previous technique that used a regenerative optical amplifier to provide pulse powers of 10–40 nJ and oil immersion, high NA short working distance objectives to break down the optical transparency and cause rapid heating and vaporization at the focal spot [49]. That previous approach had the advantage that the damage could be accomplished in less than a second with relatively few pulses of laser irradiation and showed immediate effects. Our approach required many seconds with far more pulses and required many minutes to discern the damage (although photobleaching was evident quickly). However, because we were damaging axons by virtue of their ability to absorb the multiphoton excitation, we may have achieved a degree of selectivity that would not be possible otherwise. We believe this damage depends on absorbance of the two photon excitation because there was no damage at the same power level when the laser was not mode-locked. Fluorescence excitation thus appears critical for damage at the laser intensities we used. At the powers used, no scarring or collateral damage to muscles fibers or nearby axons was observed, nor was there a visible plasma bubble. To visualize the excitation spot and its position in the optical field simultaneously on a Bio-Rad MRC1024MP microscope system, we replaced a mirror above the objective in the excitation and return light path with a dichroic mirror (Chroma 700DCSPXR), which reflected the exciting infrared light and transmitted the fluorescent emission to the eyepieces or a camera (Figure S1). A YFP filter cube (Chroma) was used in the epifluorescence light path, above the specially installed dichroic mirror. The fluorescent axons and site of laser excitation were visualized using a low-light SIT video camera (Dage-MTI Series 68). Filters were installed to block reflection of the IR laser to the camera and eyepieces. In adults, damage was easier to generate in GFP-expressing than YFP-expressing axons. We think the lower susceptibility of YFP axons to damage may be due to lower absorption of multiphoton light by YFP at 815 nm. At longer wavelengths where YFP might be a better absorber, the laser pulse energy available in our system was substantially less than 1 nJ. In pups interestingly, axons were more easily damaged than in adults. YFP expressing axons in pups were just as susceptible to damage as GFP labeled axons in adults, and the threshold intensity for damage was roughly the same for both fluors.

### Modeling

Our model of synaptic rearrangements at a neuromuscular junction is based on evolutionary graph theory [41]. The vertices of the graph represent the individual synaptic sites of the junction. In our simulation, each site is randomly assigned to one of six axons. The axons are assumed to have equal fitness; therefore, the probability of axon withdrawal is the same at all synaptic sites. The axon growth rate is assumed to be constant for all axons. A site is selected at random for axon withdrawal. We then randomly select a neighboring synaptic site. The axon innervating this neighboring site grows to take over the vacated site. Thus an axon that occupies multiple neighboring sites has a higher probability of taking over the vacated site than an axon that innervates only one neighboring site. This process of withdrawal from a randomly selected site and takeover by a randomly selected neighboring site is repeated until all the synaptic sites of the junction are innervated by the same axon. To perform our simulations we used a macro written for Matlab (The Mathworks, Inc.; Natick, MA) based on a Potts model with non-periodic boundary conditions.

## Supporting Information

Figure S1 Modified light path of a multiphoton microscope system for laser axotomy. In order to optically target single axons for removal in living mice, the sternomastoid muscle was exposed in anesthetized mice (ventral side up) on an upright microscope stage. The microscope was equipped with standard epifluorescence optics and a low-light silicon-intensified target (SIT) video camera. A short pass (<700 nm) extended reflection dichroic mirror was placed in the light path between the objective turret and the YFP (or GFP) fluorescence filter cube to prevent any of the reflected infrared light from contaminating the visible light wavelengths seen by the video camera. This allowed for simultaneous pulsed infrared laser excitation and epifluorescence visible light illumination. Scanhead zoom mirrors were used to position a diffraction limited spot of the infrared laser to a fixed point in the specimen focal plane whose position was marked on a video monitor receiving the output of the SIT camera. Using the lateral and vertical movements of the stage, the axon of interest was then moved into position. When the shutter for the pulsed laser was opened, a bright fluorescence spot due to multiphoton excitation was visible in the wide field fluorescence image but only when the pulsed laser was striking a fluorescent object—that is, a GFP- or YFP-filled axon. The laser was allowed to dwell on the axon until the fluorescence intensity disappeared and did not rapidly recover (∼0.5–1 min). As described in the text, the axon subsequently swelled and then disintegrated. The same neuromuscular junction was reimaged with confocal microscopy after re-anesthetizing the mouse one or more times over the next several days.
(TIF)

Figure S2 Wavelength selective photobleaching of cytoplasmically expressed fluorescent protein does not damage motor neurons in vivo. (A) Natural color variation between axons in the sternomastoid muscle of a double transgenic P7 mouse pup constitutively expressing both CFP (green) and YFP (red) in all motor neurons. (B) The color difference between inputs to the topmost neuromuscular junction was enhanced by scanning with high intensity 514 nm laser light at high scan zoom at the site of the circle to photobleach the YFP in left input to this junction. (C) Both CFP and YFP were then photobleached by exciting with both 440 nm and 514 nm laser light, allowing the territory occupied by the non-bleached axon to be observed unambiguously. (D) Fluorescence recovery occurred within a few minutes with no apparent immediate damage to the irradiated input. (E) On the following day, the non-irradiated input had lost synaptic territory and the previously irradiated input has gained synaptic territory. (F) The YFP in the now-dominant input was photobleached again using 514 nm light to reveal the synaptic territory occupied by the non-irradiated input, which confirmed its loss of territory (arrow). Scale bar, 20 µm.
(TIF)

Video S1 Approximately 1,000 frame simulation showing the synaptic rearrangement process at a single neuromuscular junction. There are initially six axons. Each axon is labeled a different color. The postsynaptic receptors are not shown and assumed to be unchanging during the course of the simulation. At each frame a synaptic contact is randomly selected for withdrawal and one of the axons adjacent to the vacated site is randomly selected to grow and occupy the vacated site. Note the rapid loss of inputs early in the simulation process (first half) and the prolonged period of dual innervation (second half). There is also evidence of progressive segregation of contacts [29], flip-flop [4], and ultimately the transition to single innervation.
(MOV)

## Abbreviations

AChR: acetylcholine receptor
CFP: cyan fluorescent protein
GFP: green fluorescent protein
SIT: silicon-intensified target
YFP: yellow fluorescent protein
