# Supplementary figures and images for: Reversing the Outcome of Synapse Elimination at Developing Neuromuscular Junctions In Vivo: Evidence for Synaptic Competition and Its Mechanism
Source: PLoS Biol. 2012 Jun 26;10(6):e1001352. doi: 10.1371/journal.pbio.1001352 (PMC3383738; doi:10.1371/journal.pbio.1001352)

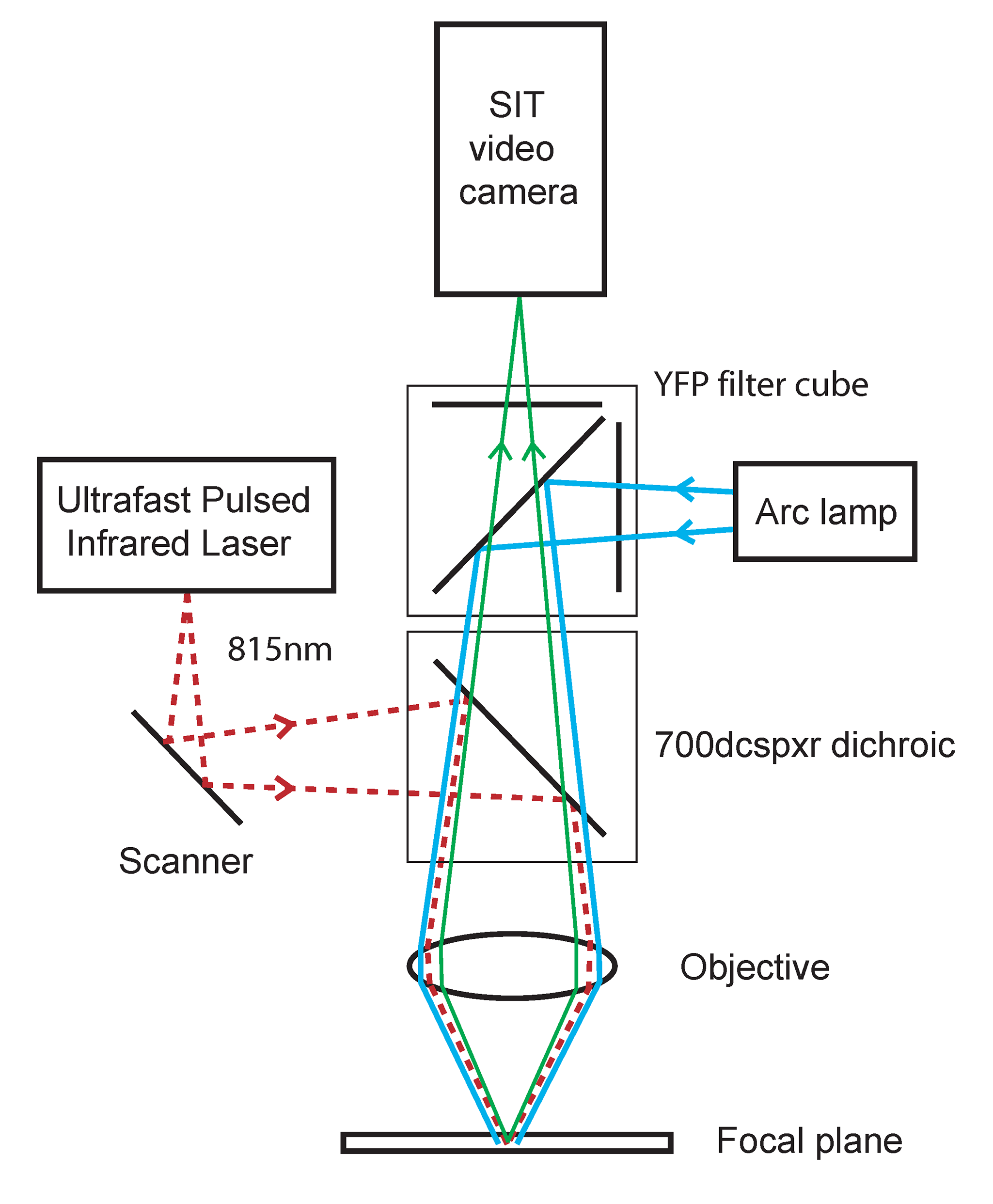

Supplement: Figure S1 — Modified light path of a multiphoton microscope system for laser axotomy. In order to optically target single axons for removal in living mice, the sternomastoid muscle was exposed in anesthetized mice (ventral side up) on an upright microscope stage. The microscope was equipped with standard epifluorescence optics and a low-light silicon-intensified target (SIT) video camera. A short pass (<700 nm) extended reflection dichroic mirror was placed in the light path between the objective turret and the YFP (or GFP) fluorescence filter cube to prevent any of the reflected infrared light from contaminating the visible light wavelengths seen by the video camera. This allowed for simultaneous pulsed infrared laser excitation and epifluorescence visible light illumination. Scanhead zoom mirrors were used to position a diffraction limited spot of the infrared laser to a fixed point in the specimen focal plane whose position was marked on a video monitor receiving the output of the SIT camera. Using the lateral and vertical movements of the stage, the axon of interest was then moved into position. When the shutter for the pulsed laser was opened, a bright fluorescence spot due to multiphoton excitation was visible in the wide field fluorescence image but only when the pulsed laser was striking a fluorescent object—that is, a GFP- or YFP-filled axon. The laser was allowed to dwell on the axon until the fluorescence intensity disappeared and did not rapidly recover (∼0.5–1 min). As described in the text, the axon subsequently swelled and then disintegrated. The same neuromuscular junction was reimaged with confocal microscopy after re-anesthetizing the mouse one or more times over the next several days. (TIF) [file pbio.1001352.s001.tif]

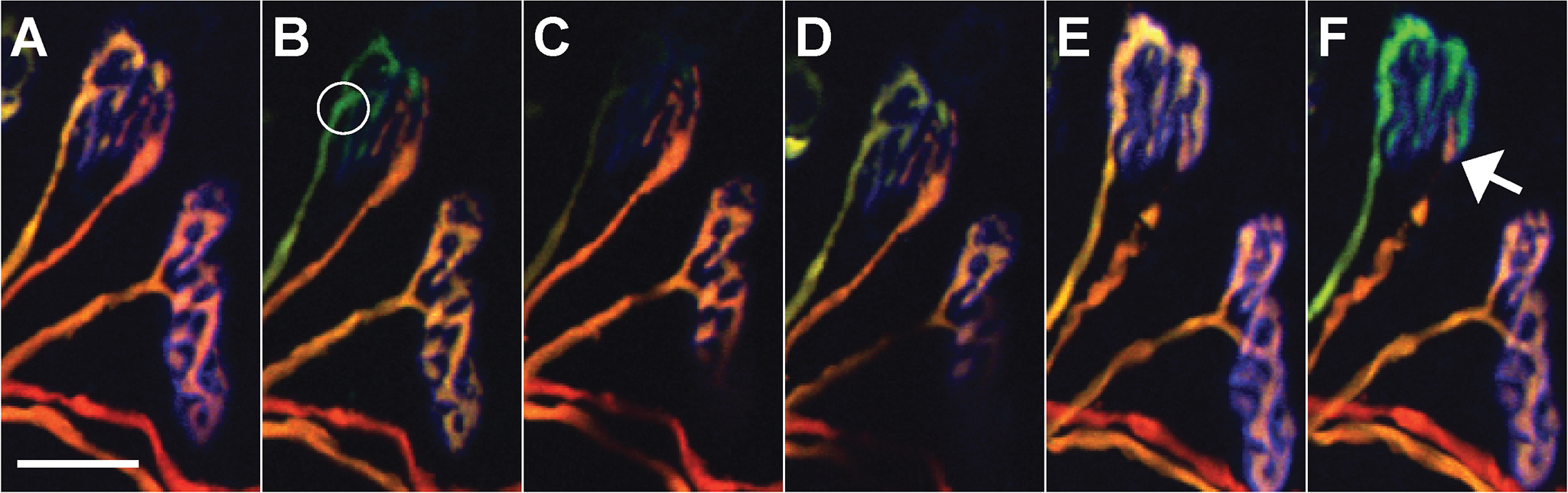

Supplement: Figure S2 — Wavelength selective photobleaching of cytoplasmically expressed fluorescent protein does not damage motor neurons in vivo. (A) Natural color variation between axons in the sternomastoid muscle of a double transgenic P7 mouse pup constitutively expressing both CFP (green) and YFP (red) in all motor neurons. (B) The color difference between inputs to the topmost neuromuscular junction was enhanced by scanning with high intensity 514 nm laser light at high scan zoom at the site of the circle to photobleach the YFP in left input to this junction. (C) Both CFP and YFP were then photobleached by exciting with both 440 nm and 514 nm laser light, allowing the territory occupied by the non-bleached axon to be observed unambiguously. (D) Fluorescence recovery occurred within a few minutes with no apparent immediate damage to the irradiated input. (E) On the following day, the non-irradiated input had lost synaptic territory and the previously irradiated input has gained synaptic territory. (F) The YFP in the now-dominant input was photobleached again using 514 nm light to reveal the synaptic territory occupied by the non-irradiated input, which confirmed its loss of territory (arrow). Scale bar, 20 µm. (TIF) [file pbio.1001352.s002.tif]
